# Isolation and Identification of a Novel Rabies Virus Lineage in China with Natural Recombinant Nucleoprotein Gene

**DOI:** 10.1371/journal.pone.0049992

**Published:** 2012-12-04

**Authors:** Cheng-Qiang He, Sheng-Li Meng, Hong-Yan Yan, Nai-Zheng Ding, Hong-Bin He, Jia-Xin Yan, Ge-Lin Xu

**Affiliations:** 1 Key Laboratory of Systems Biology in Universities of Shandong, College of Life Science, Shandong Normal University, Jinan, China; 2 Wuhan Institute of Biological Products, Wuhan, China; 3 Dairy Cattle Research Center, Shandong Academy of Agricultural Sciences, Jinan, China; University of Florida, United States of America

## Abstract

Rabies virus (RABV) causes severe neurological disease and death. As an important mechanism for generating genetic diversity in viruses, homologous recombination can lead to the emergence of novel virus strains with increased virulence and changed host tropism. However, it is still unclear whether recombination plays a role in the evolution of RABV. In this study, we isolated and sequenced four circulating RABV strains in China. Phylogenetic analyses identified a novel lineage of hybrid origin that comprises two different strains, J and CQ92. Analyses revealed that the virus 3′ untranslated region (UTR) and part of the *N* gene (approximate 500 nt in length) were likely derived from Chinese lineage I while the other part of the genomic sequence was homologous to Chinese lineage II. Our findings reveal that homologous recombination can occur naturally in the field and shape the genetic structure of RABV populations.

## Introduction

Each year, rabies causes around 55,000 deaths worldwide with 60 % of fatalities occurring in Asia [1]. China has the second highest incidence of rabies after India [2], [3]. In the last 60 years, several rabies epidemic waves have been reported in the country. Human rabies cases have decreased during the first half of the 1990 s with a low of 159 cases reported in 1996, and subsequently the number of human rabies cases increased dramatically [4], [5]. In China, 1917 and 1879 peoples were died from rabies respectively in 2010 and 2011 (http://www.moh.gov.cn/publicfiles///business/cmsresources/mohjbyfkzj/cmsrsdocument/doc14011.doc).

The causative agent of rabies, rabies virus (RABV), belongs to the family *Rhabdoviridae*, and genus *Lyssavirus*. RABV possesses a non-segmented, single-stranded, negative-sense RNA genome of approximately 12 kb, encoding five viral proteins (3′ to 5′): nucleoprotein (N); phosphoprotein (P); matrix (M) protein; glycoprotein (G); and large protein (L) [6]. Its internal helically packaged ribonucleocapsid (RNP) is composed of the genomic RNA closely associated with protein N, polymerase L, and its cofactor protein, P. The RNP complex is responsible for genomic transcription and replication within the cytoplasm of the host cell [7].

RABV have widely genetic diversity associated with geographical distribution. According to the origin of geography, available RABV can be divided into six clades in non-flying mammals [8]. RABV diversity is attributed to its high mutation since its RNA dependent RNA polymerase has no proofing function. Homologous recombination plays important role in generating genetic diversity of some viruses [9], [10]. Recombination between RNA viruses can also result in unpredictable epidemiological results, for example, polio epidemics caused by strains generated via recombination between vaccine virus strains and other endogenous enteroviruses are threatening the World Health Organization’s (WHO) polio-eradication program [11]. Although homologous recombination was considered to be rare in negative-stranded RNA virus [12], an increasing number of recombination events have been reported for negative-sense RNA viruses, such as Newcastle virus [13], [14], [15], [16], [17], human respiratory syncytial virus [18], mumps virus [12], and Ebola virus [19]. For RABV, incongruent phylogenetic relations of different genes have been found in RABV vaccine strains [20]. To the contrary, analyses of sequence deposited in GenBank reveals that two RABV isolates have apparent evidence of homologous recombination events [21]. Nevertheless, the solitary mosaic RABV isolates available in public databases are not be robust evidence that homologous recombination can occur naturally in RABV since some recombinant viruses from GenBank were found to be likely generated artificially due to laboratory contamination, such as influenza virus [22] and Newcastle disease virus [23]. And thus, it is unknown whether homologous recombination can truly take place in RABV.

In this study, we isolated and sequenced four RABV isolates circulating in China, and identified a novel RABV lineage with an *N* gene generated by recombination between viruses from two distinct lineages circulating in China. This finding might provide important insights into the contribution of recombination in shaping the genetic diversity of RABV.

## Results

**Figure 1:**
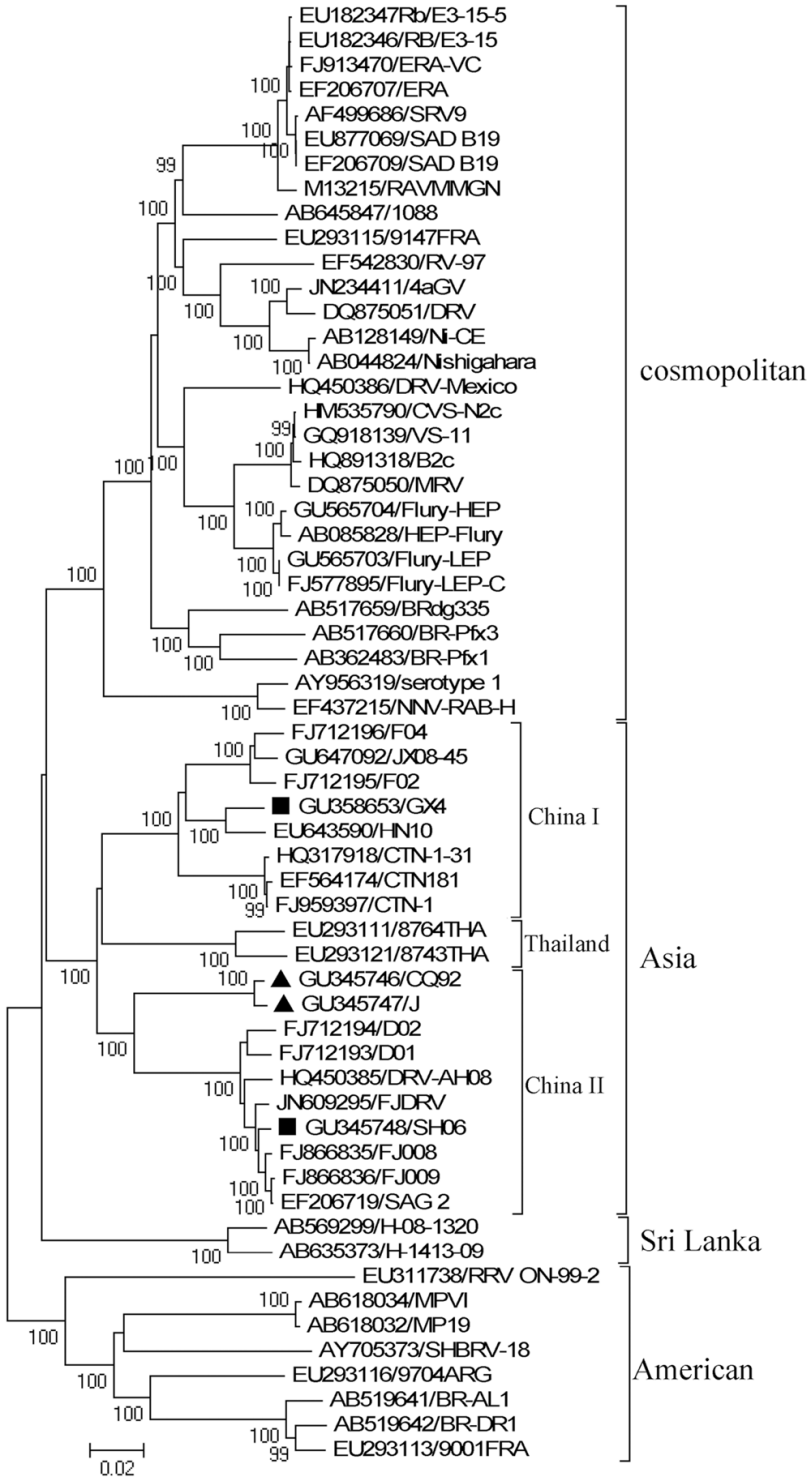
The evolutionary history of rabies virus based on complete genome sequences inferred using the neighbor-joining method. The bootstrap consensus tree was inferred from 1000 replicates and was used to represent the evolutionary history of the taxa analyzed. Branches corresponding to partitions reproduced in <50% bootstrap replicates are collapsed. The percentage of replicate trees in which the associated taxa clustered together in the bootstrap test (1000 replicates) is shown next to the branches. The phylogenetic tree was drawn to scale, with branch lengths in the same units as those of the evolutionary distances used to infer the phylogenetic tree. The evolutionary distances were computed using the Kimura 2-parameter method and are in the units of the number of base substitutions per site.

**Figure 2:**
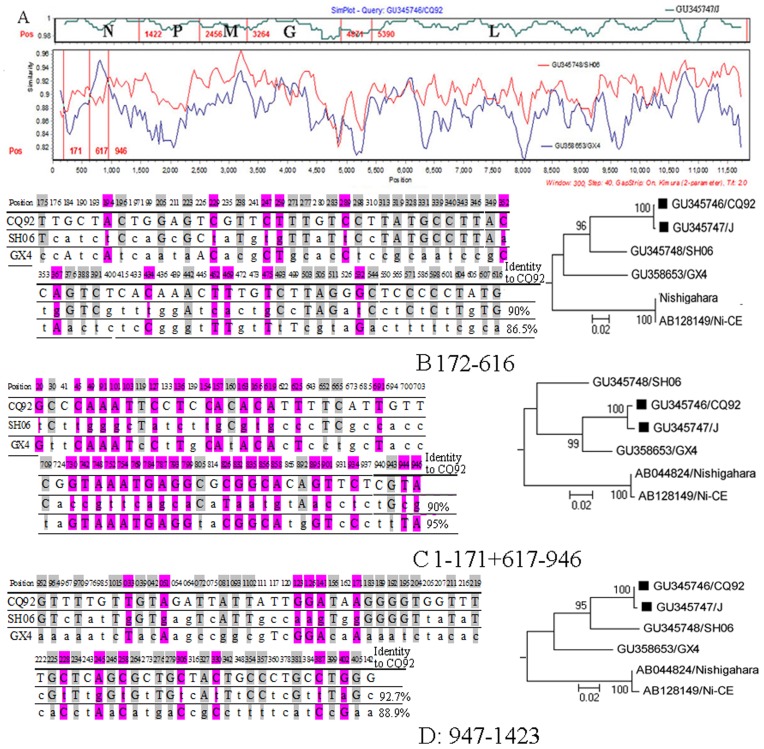
Sequence comparisons of J, CQ92, GX4 and SH06 rabies virus isolates. (A) A comparison of the genomic sequence of RABV isolates J, CQ92, GX4 and SH06. Strain CQ92 was used as the query. The y-axis gives the percentage of identity within a sliding window of 300 bp centered on the position plotted, with a step size between plots of 20 bp. The left side of (B), (C) and (D) panels showed the variant sites of CQ92 and its parent lineage representatives GX4 and SH06 in different genomic regions. The identity between CQ92 and its parent lineages is also shown in the middle of each panel. The variant sites identical to GX4 and SH06 are shaded in pink and grey, respectively. The right side of each panel shows the analysis of homology between CQ92 and parental lineages to show the different origins of CQ92. The phylogenetic histories of each region were inferred using the neighbor-joining method with a Tamura 3-parameter model. The robustness of each tree was tested by bootstrapping with 1000 replicates. Bootstrap values are shown beside the branches. (B) Positions 172–616. (C) Positions 1–171 and 617–941. (D) Positions 942–1423.

Four RABV strains were isolated and their complete genomes were sequenced. Each viral strain was sequenced several times to exclude the possibility of laboratory contamination. After the all genomic sequences of global RABV were aligned, Xia’s test was performed and suggested there was not mutation saturation in the RABV sequence alignment file (Figure S1). Similar to a previous report [8], our phylogenetic analysis grouped these RABV genomic sequences into four different clades with robust bootstrap support associated with geographic origin. (Figure 1). The four strains isolated in this study were shown to be most homologous with the Asia group, comprised of three different lineages, China I, China II, and Thailand. While RABV strains J, CQ92, and SH06 grouped into the China II group, GX4 clustered within the China I group. Moreover, J and CQ92 constituted a single branch within the China II group with significant bootstrap support (Figure 1). Genomic sequence comparisons showed that J and CQ92 shared very high sequence identity (99.18%) (Figure 2A Upper). Interestingly, CQ92 shared higher sequence identity with GX4 than SH06 from positions 1 to 171 and 617 to 946 (95% *vs.* 90%) but had higher sequence identity with SH06 than GX4 in other regions (Figure 2A Lower). Phylogenetic analysis also showed that strains CQ92 and J cluster together in the same lineage with GX4 (Figure 2C right) for the former two regions. These data suggest that strains CQ92 and J might be recombinant strains descended from both China I and II lineages.

According to the recombination analysis tools RDP (version 3.0) [24], seven programs determined that the two strains had significant recombination signals: RDP [25], p-value <0.05; Geneconv [26], p-value <0.001; Bootscan [27], p-value <0.005; Maxchi [28], p-value <0.005; Chimaera [29], p-value <0.05; Siscan [30], p-value <0.001; and 3Seq [31], p-value <0.05 (Table 1). In addition, the phi test implemented in SplitTrees software package [32] did also find statistically significant evidence for recombination (p = 0.029) in *N* gene. Therefore, we proposed that CQ92 and J strains should be originated from recombination between the two lineages circulating in China.

**Table 1:** Recombination confirmation table of different recombination analysis methods.

Methods	Av. P-value
RDP	1.76×10^−2^
Bootscan	1.778×10^−3^
Geneconv	9.87×10^−4^
Maxchi	2.48×10^−3^
Chimaera	4.37×10^−3^
Siscan	1.76×10^−2^
3Seq	2.59×10^−2^

**Figure 3:**
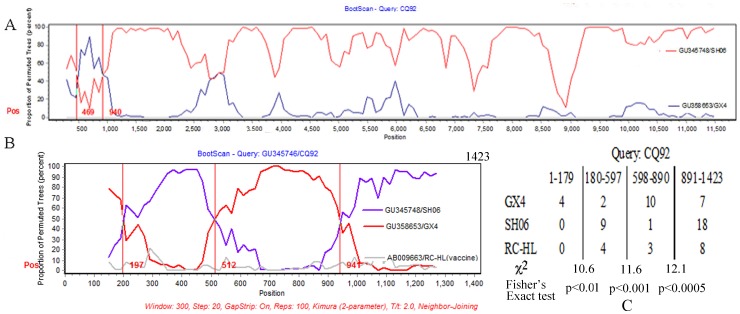
Analysis of recombination breakpoints. (A) The bootscan result of CQ92, GX4 and SH06 complete genome. RC-HL was used as the outgroup. The window size was set at 600 bp to avoid noise from gene mutations. (B) The bootscan result of region from positions 1–1423. The parameters used for analysis are shown on the bottom row of the figure. The window size was set at 300 bp. The three crossover sites are represented by vertical lines. (C) The statistical analysis of informative sites. Vertical lines represent the recombination breakpoints with the maximization of χ^2^. χ^2^ of each breakpoint and *P*-value of Fisher’s exact test are shown under the vertical lines.

The recombination analysis package, Simplot, was used to further analyze and determine the genomes of CQ92 and J for putative recombination events in order to identify the potential breakpoints [33]. First, the Bootscan program was used to determine if there indeed was evidence of a recombination event. To avoid mutational noise, the size of sliding window and step width was respectively set at 600 bp and 40 bp. The bootscan result of complete genome of CQ92 showed that >70% of permuted trees of CQ92 were homologous to the GX4 lineage from positions 469 to 940 (Figure 3A). However, if the sliding window was shortened to 300 bp, three crossover sites were located around positions 197, 512, and 941 (Figure 3B). The Simplot and Bootscan analysis of different window sizes were also shown in Figures S2 and S3. Employing the Findsites algorithm implemented in Simplot program, statistical analysis of informative sites was performed using RC-HL as an outgroup (Figure 3C). The maximum values of χ^2^ were found at positions 179, 597, and 890 (10.6, 11.6, and 12.1, respectively; *P*-value of Fisher’s Exact test <0.01).

**Figure 4:**
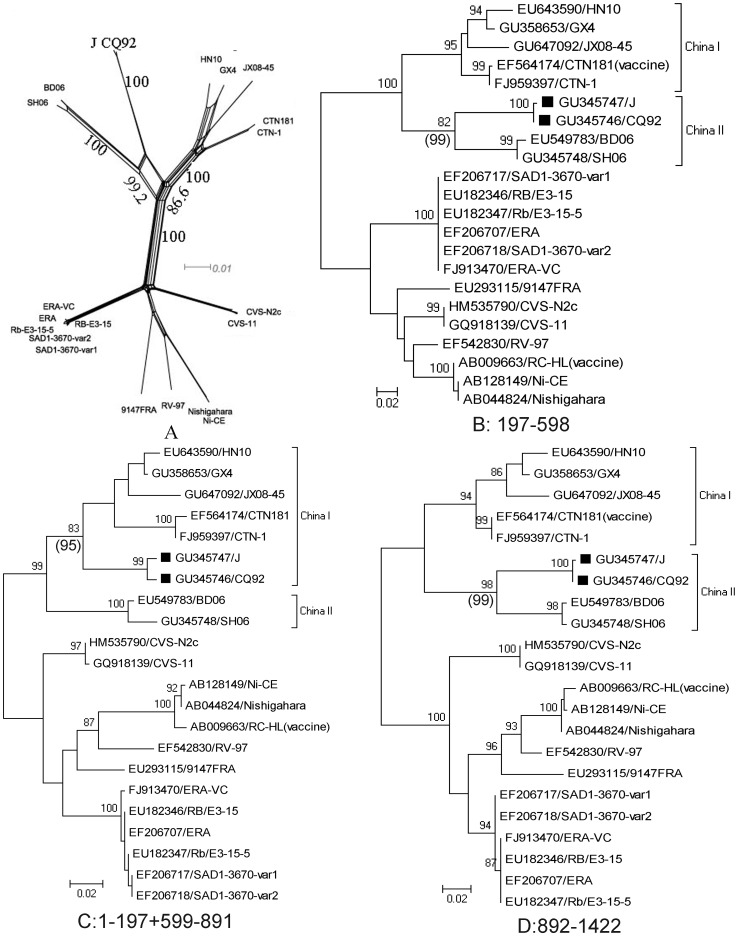
Analysis of the origin of the CQ92 lineage in different regions of the *N* gene delimited by the putative breakpoints. (A) A split tree inferred from the complete *N* gene sequence showing the evolutionary relationship. A networked pattern of mosaic lineage was found to be related to lineages GX4 and SH06. The Neighbor-Net tree was constructed by employing the SplitsTree4 program. (B) Phylogenetic relationships from positions 180 to 598 of CQ92 genome. (D) Phylogenetic relationship of the *N* gene fragment from positions 1–179 and 599–891. (D) Phylogenetic relationship of positions 892–1423. The evolutionary history of each fragment was inferred using the maximum likelihood method with the Kimura 2-parameter substitution model and Neighbor-Joining (NJ) method with the Maximum Composite Likelihood model. The percentage (>80%) of replicate ML trees in which the associated taxa clustered together in the bootstrap test (1000 replicates) is shown beside the branches. The NJ tree bootstrap values of the branch associated with recombinant strain are shown in parentheses. The tree is drawn to scale, with branch lengths shown in the same units as those of the evolutionary distances used to infer the phylogenetic tree. ▪, mosaics.

To further analyze the identified recombination event, several representative sequences of the Chinese lineages and the cosmopolitan group (listed in Figure 4) were used to construct a split tree using a sequence alignment before position 1423. Strains J and CQ92 constituted a separate branch with 100% bootstrap (1000 replicates) and showed a networked evolutionary pattern associated with Chinese lineage I (with 86.6% bootstrap value of 1000 replicates) and Chinese lineage II (99.2% bootstrap value) (Figure 4A), suggesting that the *N* gene of J and CQ92 was a mosaic of these two Chinese lineages.

A set of statistically incongruent phylogenetic trees is thought to be the gold-standard method to demonstrate the presence of recombination [34]. If bootstrap support on the tree indicates that the recombinant sequences cluster with one parent group for one sequence region and another parent group for another sequence region, this is considered as a statistically-supported phylogenetic recombination signal [34]. Employing MEGA 5, maximum likelihood (ML) phylogenetic analyses showed that J and CQ92 clustered into different lineages at different regions delimited by the putative breakpoints (Figures 4B–E). From positions 1–179 and 598–891, J and CQ92 were clustered into the GX4 lineage with bootstrap values of >80% (Figures 4C). However, the two mosaics were nested in the SH06 lineage in complementary regions with >85% bootstrap value (Figure 4B, 4D). Therefore, these incongruent phylogenetic trees provided reliable evidence that homologous recombination likely occurred in the *N* gene of both strains J and CQ92.

These results showed that J and CQ92 belonged to a recombinant lineage descended from China I and II lineages of RABV Asia group. The 3′ untranslated region (3′-UTR) and part of the *N* gene likely originated from Chinese lineage I and the remainder of the genome was derived from Chinese lineage II. The length of the two fragments descended from Chinese lineage I was determined to be approximate 500 nucleotides in total.

We observed a significant difference at four amino acid sites (D11N, S42T, S228T, and R263K) between GX4 and SH06 in recombination regions. Therefore, the exchange of genetic material between the parent lineages might also bring about the rapid change of the N protein primary structure. Homologous recombination could thus result in amino acid rapid change of N protein.

## Discussion

To keep their original characteristics, all RABV strains isolated in this study were only passaged once after purification by limiting dilution. PCR products were directly sequenced rather than cloned into plasmid vectors to sequence. Each recombination region of J and CQ92 was sequenced twice and the same recombination signals were obtained. Complete genomic sequence analysis also gave no sign of co-infection (Figure 2A). These data suggest that the recombination event in J and CQ92 was authentic.

Among 66 informative sites, 60 were located at the third site of codon (Figure 3C) and which suggests that selective pressure did not play an important role in the evolution of the *N* gene. It is unlikely that selective pressure could cause 35 of 60 variant sites (Figure 2C) in recombination regions of approximate 500 nucleotides length to directly change from Chinese lineage II to Chinese lineage I. Therefore, the mosaic signal in the CQ92 lineage is likely to be a result of homologous recombination rather than selective pressure of convergent evolution.

The two strains isolated in this study, J and CQ92, were obtained at different dates (1985 and 1992, respectively) and from different locations (Chongqing city and Ningxia Province, respectively) approximately 1500 km apart. However, since the genomic sequences of the two strains shared very high identity (99.2%) (Figure 2), and exhibited the same recombination pattern, it is highly unlikely that the two recombinants were generated from two distinct recombination events. This is the first direct and conclusive evidence supporting natural homologous recombination in RABV.

Genetic typing based on *N* gene sequences has been widely used to determine the genotype and trace the distribution and spread of RABV. Here we report that recombination can occur within the *N* gene and studies that ignored the possibility of recombination might compromise these phylogenetic analyses since different genomic regions will have different evolutionary histories [27]. Therefore, intragenic recombination must be excluded before phylogenetic analyses to decipher the molecular epidemiology of RABV.

Several street Alabama Dufferin (SAD)-derived vaccine strains of RABV have been reported to undergo recombination [20]. Retrospective analyses revealed that two isolate sequences available in GenBank had evidence of recombination [21]. The current study demonstrates that recombination can occur naturally in the field that resulted in a prevailing RABV lineage. These studies suggest that homologous recombination might be an important evolutionary force in shaping the genetic diversity of RABV. Thus, it is possible that recombination could result in a RABV lineage with novel epidemiology. For RABV, it has two principal vectors mainly belonging to the orders *Carnivora* and *Chiroptera.* The genetic basis triggering RABV switch from bats to carnivores have not been well explained since intrinsic mutability of the virus can be insufficient to overcome phylogenetic barriers at two crucial stages of viral emergence: initial infection and sustained transmission [35]. Recombination allows viruses to acquire many key adaptive mutations in a single step, which might increase viral fitness and/or lead to changes in host tropism [36]. It is necessary to study whether recombination can result in host switch of RABV.

Comparing protein polymorphisms identified in the genomes of isolated viruses, we found that the recombination event likely resulted in an increase mutation rate leading to amino acid changes in the N protein of recombinants. Therefore, recombination might also play a potential role in shaping the adaptive evolution of RABV, which, in turn, can result in changes in the epidemiology of the virus. In fact, some virulent virus strains have been generated by homologous recombination between attenuated vaccine and wild viruses [11], [37].

In conclusion, the current study identified a RABV lineage of hybrid origin and provided strong evidence that homologous recombination can occur in RABV. Given that recombination can lead to the emergence of novel pathogens with unpredicted epidemiological results, RABV recombinants should receive appropriate attention in future RABV surveillance.

## Materials and Methods

### Viruses

Using previously described methods [3], [38], we isolated four RABV street strains. RABV J strain was isolated from the saliva of a patient with clinical signs indicating rabies in Ningxia, China in 1985. The patient volunteered to submit the specimen for identification of RABV before dying from rabies. CQ92, GX4, and SH06 were isolated from the brain tissue of three deceased dogs with clinical signs of rabies that presented in Chongqing, Guangxi, and Shanghai, in 1992, 1994, and 2006, respectively. After purification by limiting dilution, viruses were amplified in 1–4 day old mice (Kunming white strain). After 7 days, mice displayed rabies symptoms. The presence of RABV in brain tissues was determined by enzyme-linked immunosorbent assay (ELISA) and frozen at −70°C before RNA isolation. To keep their original characteristics, all RABV strains were passaged only once after purification and sequencing. This study was approved by the Review Board of Shandong Normal University, China. All collection of samples and experiments were performed in accordance with the Chinese animal protection laws.

### RNA Extraction and Sequencing

Viral RNA was extracted from approximately 0.1 g of virus cultures using 1 mL TRIzol (Invitrogen, Carlsbad, CA, USA), following the manufacturer’s instructions. Reverse transcription was performed using random hexamer primers and the complete genome was sequenced. Amplification of the *N* genes was performed by reverse transcript polymerase chain reaction (RT-PCR) using the Ex-Taq (TaKaRa Bio, Otsu, Shiga, Japan) enzyme in a Perkin Elmer Cetus DNA Thermal Cycler using the following primers: forward primer, RABV1-F (5′-GTACCTAGACGCTTAACAAC-3′, positions 1–11 of PV strain); reverse primer, RHN-S8 (5′-AGGGAGACTGTCCACTTCTATAGG-3′, positions 1641–1664 of PV strain). PCR products were directly sequenced using an Applied Biosystems 3770 DNA automated sequencer (Applied Biosystems, Foster City, CA, USA). The primers RABV1-F, N127 (5′-ATGTAACACCTCTACAATGG-3′, positions 55–74 of PV strain) and RHN-S8 were used to perform bidirectional sequencing. The sequences of long overlapping regions (positions 84–457, 373 bp; and positions 695–1055, 360 bp) in sequencing reactions were used to determine whether sequencing products of each virus culture were from a mixed sample or a single isolate. Isolated sequences have been deposited in GenBank (Accession Nos.: GU345747, GU345746, GU358653, and GU345748).

### Recombination Analysis

Identification methods of recombinants were described as previous report [42]. In brief, including our four strains, all 74 RABV genome sequences were obtained from GenBank for molecular comparisons. The nucleotide alignment file of complete genomes is available online (http://user.qzone.qq.com/1530879254/blog/1331826785#app=2). The 17 sequences of SAD and ERA vaccine derivatives were discarded from the alignments when phylogenetic analyses were performed. All RABV reference sequences were aligned using the CLUSTALW program [39]. Xia’s test was performed to measure substitution saturation of the RABV sequence alignment file [40]. The recombination analysis tools, SimPlot software [33] and RDP 3.0 [24] were employed to find the potential recombinants.

To determine potential recombination event, split phylogenetics trees were constructed utilizing the SplitTrees software package (version 4.9) [32]. The relative parameters of Networks are: Characters, Uncorrected_P; Distances, NeighborNet; Splits, EqualAngle. In addition, phylogenetic history analysis was performed to infer the homology of RABV employing Maximum likelihood (ML) and Neighbor-Joining (NJ) methods implemented in MEGA 5 [41]. The Best-Fit substitution model was used for ML analysis according to Bayesian Information Criterion within MEGA5. ML phylogenetic trees were constructed using the nucleotide substitution model of Kimura 2-parameter model and gamma distributed 4 (Γ4). Maximum Composite Likelihood model was used to Neighbor-Joining analysis. The robustness of homology was tested using bootstrap method. Bootstrap value >80% of 1000 replicates was considered as significance.

## Supporting Information

Figure S1 **The analysis of substitution saturation of RABV sequence alignment file.** A. Xia’s test. B. transition/transversion vs. divergence plot.
(DOC)

Figure S2 **Simplot analysis of different window sizes.** A. 200 bp; B. 400 bp; C 500 bp; D 600 bp.
(DOC)

Figure S3 **Bootscan analysis of different window sizes.** A. 200 bp; B. 400 bp; C 500 bp; D 600 bp.
(DOC)
