# Supplementary material for: Isolation and Identification of a Novel Rabies Virus Lineage in China with Natural Recombinant Nucleoprotein Gene
Source: PLoS One. 2012 Dec 4;7(12):e49992. doi: 10.1371/journal.pone.0049992 (PMC3514186; doi:10.1371/journal.pone.0049992)
Supplement: Figure S1 — The analysis of substitution saturation of RABV sequence alignment file. A. Xia’s test. B. transition/transversion vs. divergence plot. (DOC) [file pone.0049992.s001.doc]

### Figure S1


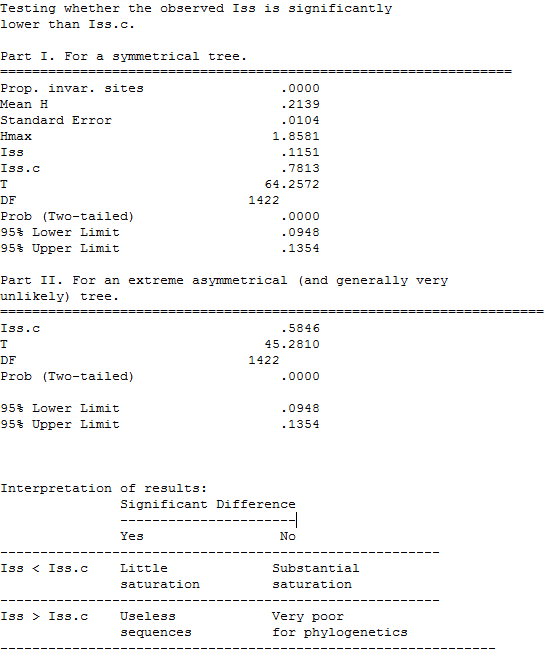


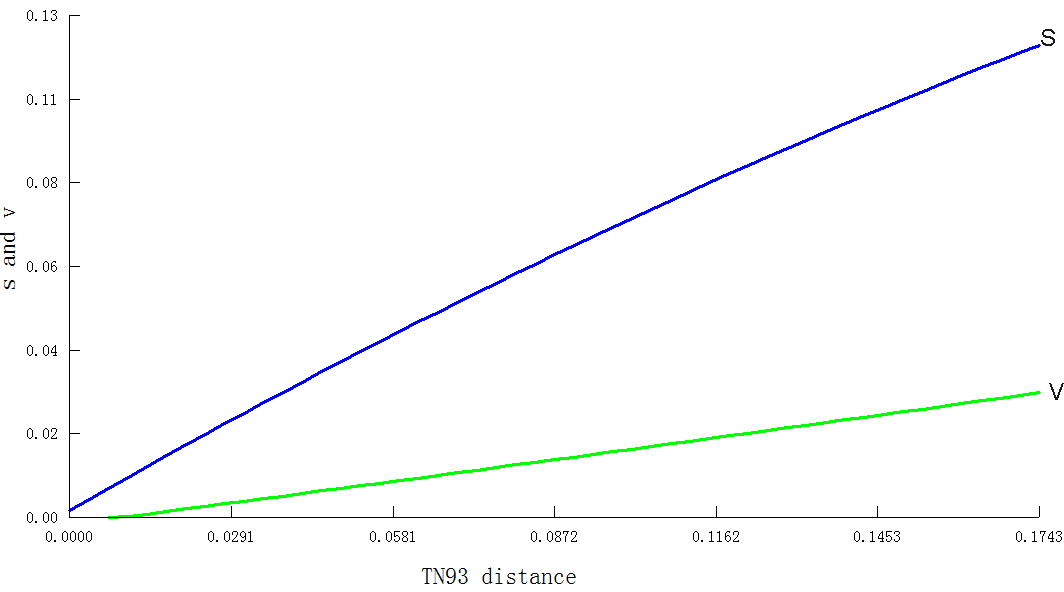


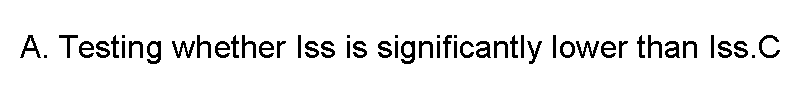

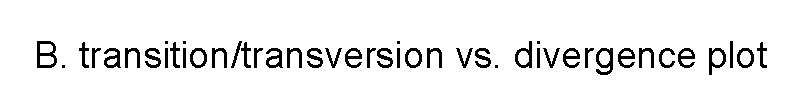


The analysis of substitution saturation of RABV sequence alignment file. A. Xia’s test. B. transition/transversion vs. divergence plot.
