# Supplementary figures and images for: Isolation and Identification of a Novel Rabies Virus Lineage in China with Natural Recombinant Nucleoprotein Gene
Source: PLoS One. 2012 Dec 4;7(12):e49992. doi: 10.1371/journal.pone.0049992 (PMC3514186; doi:10.1371/journal.pone.0049992)

### Figure S2


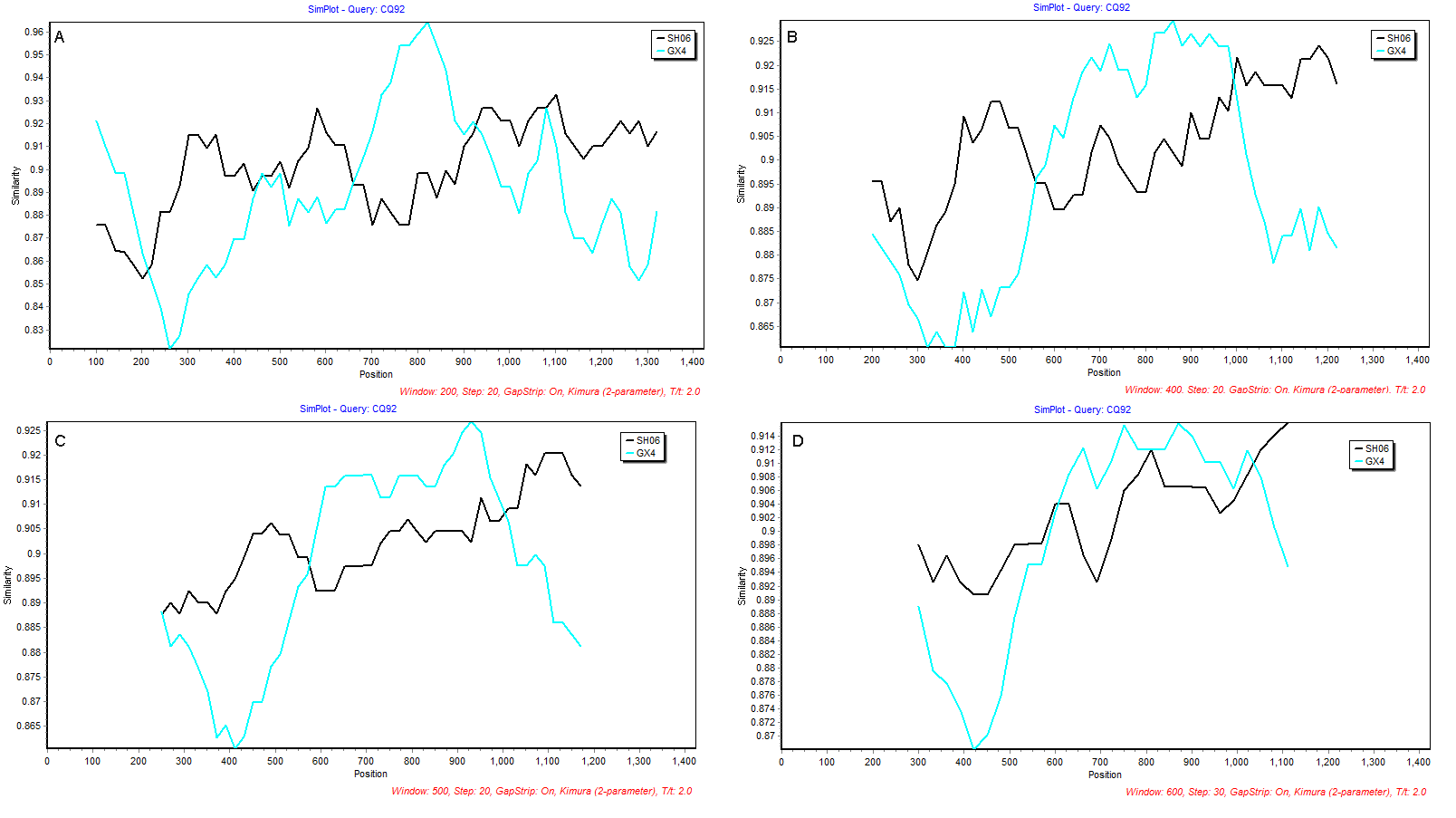


Simplot analysis of different window sizes. A. 200 bp; B. 400 bp; C 500 bp; D 600bp.

Supplement: Figure S2 — Simplot analysis of different window sizes. A. 200 bp; B. 400 bp; C 500 bp; D 600 bp. (DOC) [file pone.0049992.s002.doc]

###
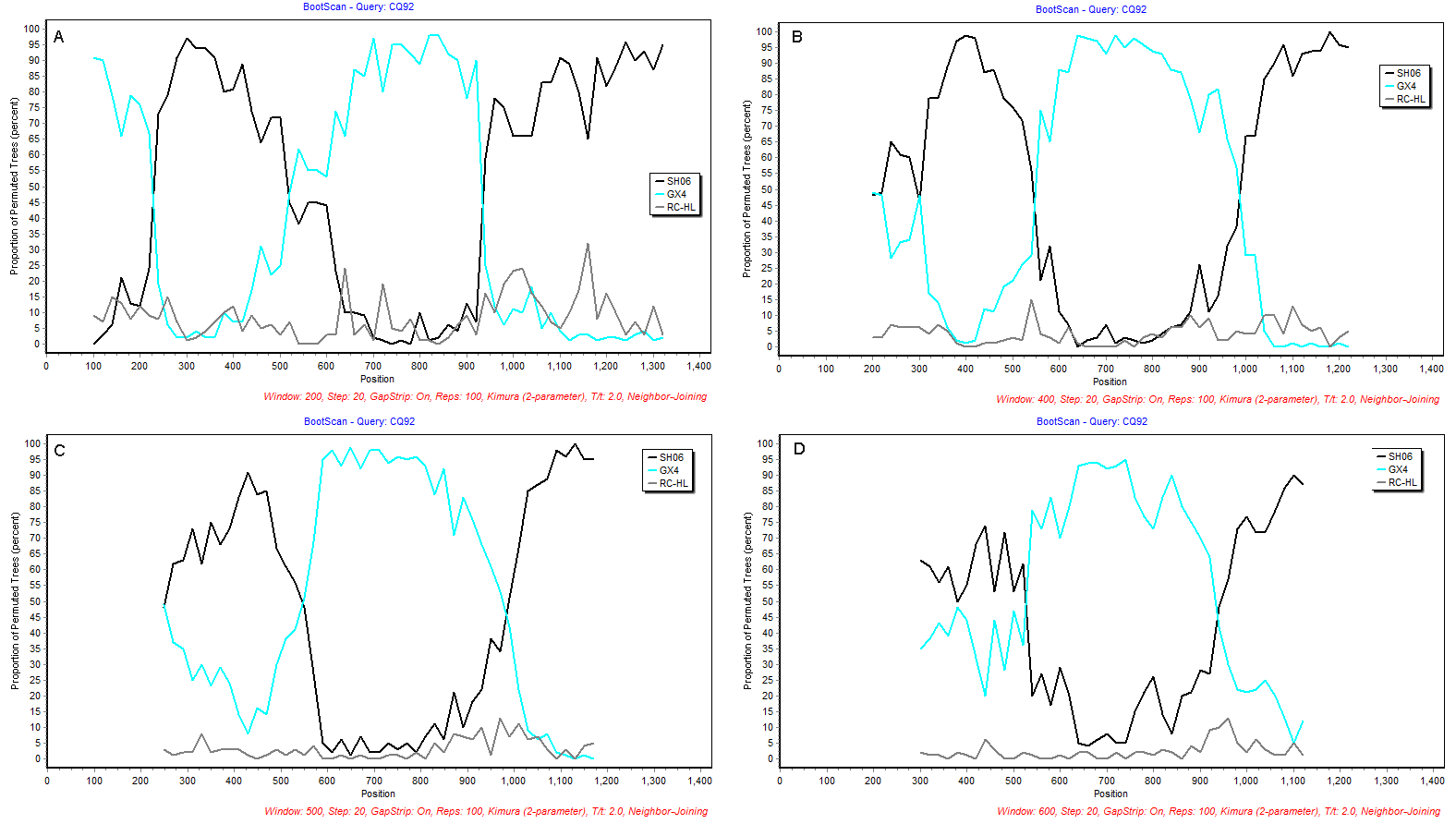
Figure S3

Bootscan analysis of different window sizes. A. 200 bp; B. 400 bp; C 500 bp; D 600bp.

Supplement: Figure S3 — Bootscan analysis of different window sizes. A. 200 bp; B. 400 bp; C 500 bp; D 600 bp. (DOC) [file pone.0049992.s003.doc]
